# MicroRNA-1915-3p inhibits cell migration and invasion by targeting SET in non-small-cell lung cancer

**DOI:** 10.1186/s12885-021-08961-8

**Published:** 2021-11-13

**Authors:** Hongli Pan, Zhenhua Pan, Fengjie Guo, Fanrong Meng, Lingling Zu, Yaguang Fan, Yang Li, Mengjie Li, Xinxin Du, Xiuwen Zhang, Yi Shao, Mingming Wei, Xuebing Li, Qinghua Zhou

**Affiliations:** 1 Tianjin Key Laboratory of Lung Cancer Metastasis and Tumor Microenvironment, Tianjin Lung Cancer Institute, Tianjin Medical University General Hospital, Tianjin, China; 2 Tianjin Prenatal Diagnostic Center, Obstetrics and Gynecology Department, Tianjin Medical University General Hospital, Tianjin, China; 3 Sichuan Lung Cancer Center, West China Hospital, Sichuan University, Chengdu, China; 4 Department of Lung Cancer Surgery, Tianjin Medical University General Hospital, Tianjin, China; 5 Department of Thoracic Surgery, Yizheng People’s Hospital, Yangzhou, Jiangsu Province China; 6 Department of Oncology, Tianjin Medical University General Hospital, Tianjin, China; 7 The State Key Laboratory of Medicinal Chemical Biology, College of Pharmacy, Nankai University, Tianjin, China

**Keywords:** NSCLC, miR-1915-3p, Invasion, Migration, SET

## Abstract

**Background:**

MicroRNAs (miRNAs) have been reported to play significant roles in non-small-cell lung cancer (NSCLC). However, the roles of microRNA (miR)-1915-3p in NSCLC remain unclear. In this study, we aimed to explore the biological functions of miR-1915-3p in NSCLC.

**Methods:**

The expression of miR-1915-3p and SET nuclear proto-oncogene (SET) in NSCLC tissues were examined by quantitative real-time PCR (qRT-PCR). Migratory and invasive abilities of lung cancer were tested by wound healing and transwell invasion assay. The direct target genes of miR-1915-3p were measured by dual-luciferase reporter assay and western blot. Finally, the regulation between METTL3/YTHDF2/KLF4 axis and miR-1915-3p were evaluated by qRT-PCR, promoter reporter assay and chromatin immunoprecipitation (CHIP).

**Results:**

miR-1915-3p was downregulated in NSCLC tissues and cell lines, and inversely associated with clinical TNM stage and overall survival. Functional assays showed that miR-1915-3p significantly suppressed migration, invasion and epithelial-mesenchymal transition (EMT) in NSCLC cells. Furthermore, miR-1915-3p directly bound to the 3′untranslated region (3′UTR) of SET and modulated the expression of SET. SET inhibition could recapitulate the inhibitory effects on cell migration, invasion and EMT of miR-1915-3p, and restoration of SET expression could abrogate these effects induced by miR-1915-3p through JNK/Jun and NF-κB signaling pathways. What’s more, miR-1915-3p expression was regulated by METTL3/YTHDF2 m6A axis through transcription factor KLF4.

**Conclusions:**

These findings demonstrate that miR-1915-3p function as a tumor suppressor by targeting SET and may have an anti-metastatic therapeutic potential for lung cancer treatment.

## Background

Lung cancer is the second most frequent cancer and the leading cause of cancer-related fatality worldwide, with 2.21 million new cases (approximately 11.4% of total) and 1.80 million deaths (almost 18.0% of total) estimated in 2020 [1]. Approximately 85% of lung cancers is non-small-cell lung cancer (NSCLC), associated with a poor prognosis [2]. Although extensive research and progress in the early diagnosis and targeted therapies of lung cancer have a rapid development in recent decades, metastasis is still the main challenge posed by advanced lung cancer resulting in a high mortality [3]. Previous studies have verified that more than 50% of NSCLC patients present with metastasis [4]. Therefore, further investigations should focus on the molecular mechanism of NSCLC metastasis.

MicroRNAs (miRNAs) are a kind of small endogenous non-coding RNAs with 18–25 nucleotides in length, which can alter mRNA stability and/or translation efficiency of target genes by perfect or imperfect base-pairing with the 3′untranslated regions (UTRs) [5], 5′UTRs [6] or open reading frames (ORFs) region [7] of target mRNAs. Accumulating evidence have shown that miRNAs participate in a wide range of biological processes in several types of cancers [8–10]. In NSCLC, many miRNAs have been proved to function as oncogenes or tumor suppressors. For instance, miR-21, acting as an oncogene, suppressed cell apoptosis and promoted NSCLC tumorigenesis through inhibition of negative regulators of the Ras/MEK/ERK pathway in vitro and in vivo [11]. In contrast, miR-34 was downregulated in NSCLC and modulated the expression of proteins involved in cell cycle, anti-apoptosis, tumor metastasis and immune invasion [12, 13], served as a tumor suppressor. Moreover, several studies revealed that miRNA mimics or inhibitors have potential as anticancer therapeutics [14, 15]. However, only few miRNA therapeutics have so far moved into clinical development. The absence of tumor-specific miRNA therapeutic options for NSCLC underscores the critical need to achieve a better understanding of the biological roles of miRNAs in lung cancer, thus developing better therapeutic strategies for NSCLC patients.

In our previous research, in order to achieve a better understanding of the role of miRNAs in lung cancer, we conducted miRNA microarray in 10 paired NSCLC tissues and found that miR-1915-3p was one of the most significantly downregulated miRNAs in NSCLC tissue samples [16]. Several studies have shown that miR-1915-3p modulates multidrug resistance, anti-apoptosis and stem cell differentiation by targeting BCL2 apoptosis regulator (BCL2) [17–19], prominin 1 (PROM1) and paired box 2 (PAX2) [20] in multiple types of cancers. Although several miR-1915-3p’s target genes have been confirmed, the underlying mechanisms of the involvement of miR-1915-3p and its targets in NSCLC metastasis are still not fully understood.

In this study, we investigated whether miR-1915-3p expression was associated with clinicopathological parameters, whether miR-1915-3p modulated the metastatic abilities of NSCLC cells by altering its targets expression, and whether miR-1915-3p expression was regulated by N^6^-methyladenosine (m6A) modification.

## Methods

### Patients and tissue specimens

This study was approved by the ethics committee of Tianjin Medical University General Hospital (Tianjin, China). In our study, we used 73 paired NSCLC tissues. We also obtained written informed consent from the 73 NSCLC patients. All of these patients were not received any chemotherapy or radiotherapy before surgery. Tumor and corresponding normal lung tissue samples were taken at the time of surgery and rapidly frozen in liquid nitrogen. The tumor tissues contained a tumor cellularity of greater than 60% and the matched control tissues had no tumor content. The clinicopathological characteristics of all patients were collected and analyzed.

### Cell culture and transfection

The NSCLC cell lines (A549 and H1975) were cultured in F-12 K or RPMI-1640 medium supplemented with 10% FBS (Hyclone, USA) at 37 °C and 5% CO_2_.

Negative control, miR-1915-3p mimic, anti-miR-1915-3p were synthesized by GenePharma (Shanghai, China). SiNC (cat. no. sc-37,007), siSET (cat. no. sc-43,856), siMETTL3 (cat. no. sc-92,172), siYTHDF2 (cat. no. sc-78,661) and siKLF4 (cat. no. sc-35,480) were purchased from Santa Cruz biotechnology (Dallas, Texas, USA). The sequences of these oligonucleotides were as follows: miR-NC: 5′-UUCUCCGAACGUGUCACGUTT-3′; miR-1915-3p mimic: 5′-CCCCAGGGCGACGCGGCGGG-3′; anti-miR-1915-3p: 5′-CCCGCCGCGUCGCCCUGGGG-3′; anti-miR-NC: 5′-CAGUACUUUUGUGUAGUACAA-3′.

For SET overexpression, SET were amplified from H1975 cDNA, and subcloned downstream of FLAG tag in the pcDNA3.1-flag vector. The primers used for the coding sequences of SET amplification were as follows: 5′-GCGCGAATTC (EcoR I) ATGTCGGCGCCGGCGGCCAA-3′ (forward primer) and 5′-GCGCCTCGAG (Xho I) TTAGTCATCTTCTCCTTCATCCTC-3′ (reverse primer). For human KLF4 overexpression, the plasmid FLAG-KLF4 was generated, and the primers were as follows: 5′-GCGCGAATTC (EcoR I) ACATTAATGAGGCAGCCACCTG-3′ (forward primer) and 5′-GCGCGGATCC (BamH I) CGGGGGATTTAAAAATGCCTC-3′ (reverse primer).

Transient transfection of A549 or H1975 cells were performed using Lipofectamine 2000 (Invitrogen, USA).

### Quantitative RT-PCR

The expression level of related genes was measured by quantitative real-time PCR (qRT-PCR). We used the 2^−ΔΔCT^ method to calculate the fold changes of mRNA or miRNA expression. The primers used for qRT-PCR were presented in Table 1. The primers of miR-1915-3p and RNU6B were purchased from Qiagen. The primers used for qRT-PCR were synthesized by BGI (BGI, China).

**Table 1:** The primers used for qRT-PCR

Gene	Primer (5′-3′)
**C1RL**	F: ATCTCATTCGTCGGTTCG
	R: TGGGCAGTCTTGTTCTCC
**CHTF8**	F: TCGCTACAGCACTGGATTA
	R: GGACGCTGGTGATAATGG
**MMP24**	F: GGCAAAAACACATCACCTAC
	R: GGTCACTTTTGATCTCATGG
**PPP2R5D**	F: CATCTCTTCCCTTTCCTTCATTCG
	R: AGCCACTGGAACAAGACAATCC
**SET**	F: CGAAGTCCACCGAAATCAAATGG
	R: TCAGAATGGTCAGTAAACCAGGT
**TFE3**	F: TGCTCCATCCTTTGTCTTG
	R: GCTGGGTCTCATCCTCACT
**TSKU**	F: CCCGAGTAACTTATGTTCAATG
	R: GACCCGAGTCTGGTTTGG
**KLF4**	F: TCGGGCGGCTTCGTGGCCGA
	R: CGTACTCGCTGCCAGGGGCG
**METTL3**	F: CAAGCTGCACTTCAGACGAA
	R: GCTTGGCGTGTGGTCTTT
**YTHDF2**	F: TAGCCAACTGCGACACATTC
	R: CACGACCTTGACGTTCCTTT
**GAPDH**	F: GAGTCAACGGATTTGGTCGT
	R: GACAAGCTTCCCGTTCTCAG
**Primary (pri) miR-1915**	F: TGTCCCCTTCTCTCCAGCT
	R: GCTGCAGAGGCAGGGCTTTC
**Precursor (pre) miR-1915**	F: AGGCCGCACCTTGCCTT
	R: TGGGGCCCACGGGTGCA

*F* Forward, *R* Reverse

### Wound healing assay

For the wound healing assay, A549 and H1975 cells were seeded into six-well plates and grown to 100% confluence. Wounds were created in the middle of the cell monolayer and assessed by phase-contrast microscope at indicated time points. Wound width was measured from four different positions. Relative wound width was calculated as the ratio between the final and the initial actual wound width.

### Cell invasion assay

Cell invasion assay was conducted by using Matrigel Invasion Kits (Corning, USA). Transwell inserts were rehydrated for at least 2 h. Transfected cells (2 × 10^4^ cells) were resuspended in serum-free medium and then placed into the upper chamber, while complete medium containing 10% FBS was added in the bottom chamber as a chemoattractant. 24 h later, cells were fixed by methanol and stained with crystal violet. The non-invading cells on the upper surface were removed by wiping with a cotton swab. The invading cells were counted through microscope. At least five fields were counted from each chamber to assess the invasive property of cells. All assays were repeated independently in triplicates.

### 3′UTR dual-luciferase reporter assay

The 3′UTR sequences of SET including miR-1915-3p binding sites were obtained from A549 cDNA, termed SET-3UTR. SET-3UTR-Del excluding miR-1915-3p binding sites were synthetized from BGI (BGI, Shenzhen, China). SET-3UTR and SET-3UTR-Del sequences were then subcloned downstream of the firefly luciferase gene in the modified pGL3-control vector, respectively.

For dual-luciferase reporter assay, cells were co-transfected with 0.2μg of the constructed pGL3-control reporter, 20 nM of the miR-1915-3p mimic or miR-NC, and 0.02 μg of Renilla expressing plasmid as an internal control by using Lipofectamine 3000 (Invitrogen). After transfection for 48 h, luciferase activities were measured using the dual-luciferase reporter assay system (Promega, USA). The ratio between firefly and Renilla luciferase activity represented relative firefly luciferase activity.

### Promoter reporter and dual luciferase assay

A 1228 bp fragment DNA containing two KLF4 binding sites from − 1005 to + 223 relative to the transcription start sites of miR-1915 was amplified from A549 genome and subcloned into pGL3-Basic vector. Deletion reporters (Del-1, Del-2, Del-1/2) were then generated.

For promoter reporter assay, cells were transfected with miR-1915 promoter reporter or KLF4 overexpression plasmid and Renilla as internal control. After transfection for 48 h, dual luciferase assay was performed according to the manufacturer’s protocol.

### Chromatin immunoprecipitation assay

Cells (1 × 10^7^) were fixed with paraformaldehyde for 10 min, and then neutralized with Glycine for 5 min. Cells were washed and harvested with PBS, and prepared for a chromatin immunoprecipitation (ChIP) assay using Magna CHIP kit (Millipore, USA). For KLF4 CHIP, IgG CHIP were performed as control. Purified DNA was then analyzed by PCR to amplify a 215 bp fragment with the primers as follows: forward, 5′-CCTGGTCACAAAGTACGTGC-3′ and reverse, 5′-CTGAGCTGGCCGCCAGCTC-3′. The PCR products were electrophoresed on a 2.5% agarose gel and stained with ethidium bromide.

### Protein extraction and western blotting

Cells were lysed on ice in RIPA buffer (Beyotime Biotechnology, China). Approximately 20 μg protein samples was separated in 12% SDS-PAGE and electroblotted onto a nitrocellulose membrane (Pall, USA). After blocking by 5% BSA in Tris-buffered saline with 1 h, the nitrocellulose membrane was hybridized with primary antibodies against anti-SET (cat. no. 55201-1-AP) from Proteintech (Rosemont, USA); anti-E-cadherin (cat. no. 14472), Vimentin (cat. no. 5741), c-Jun (cat. no. 9165), phospho-c-Jun (Ser63) (cat. no. 91952), JNK (cat. no. 9252), phosphor-JNK (Thr183/Tyr185), NF-κB pathway antibody sampler kit (cat. no. 4888), METTL3 (cat. no. 86132) from CST (Danvers, USA); N-cadherin antibody (cat. no. A0433) from ABclonal (Wuhan, China); KLF4 antibody (cat. No. sc-393,462) from Santa Cruz biotechnology (Dallas, Texas, USA); FLAG antbody (cat. no. F1804), anti-β-actin (cat. no. A3853) from Sigma (St. Louis, USA). Blots were also incubated with HRP-linked secondary antibodies that were either anti-mouse IgG (cat. no. 7076) or anti-rabbit IgG (cat. no. 7074), and detected using GeneGnome XRQ Chemiluminescence Image System (Gene Company Limited, China). Densitometric quantification of protein bands was conducted using ImageJ software and then normalized to beta-actin.

### Statistical analysis

Experimental data were presented as mean ± standard error of mean (SEM). The Chi-square test was used to determine the correlation between miR-1915-3p expression and clinicopathological parameters. Differences between groups were estimated using Student’s t test (normal distribution data) or Wilcoxon rank sum test (non-normal distribution data). The association between miR-1915-3p and SET expression was analyzed by spearman’s rank correlation. All statistical analyses were carried out using SPSS version 22.0 software (IBM, USA).

## Results

### MiR-1915-3p is downregulated in NSCLC tissues and inversely associates with prognosis

**Fig. 1:**
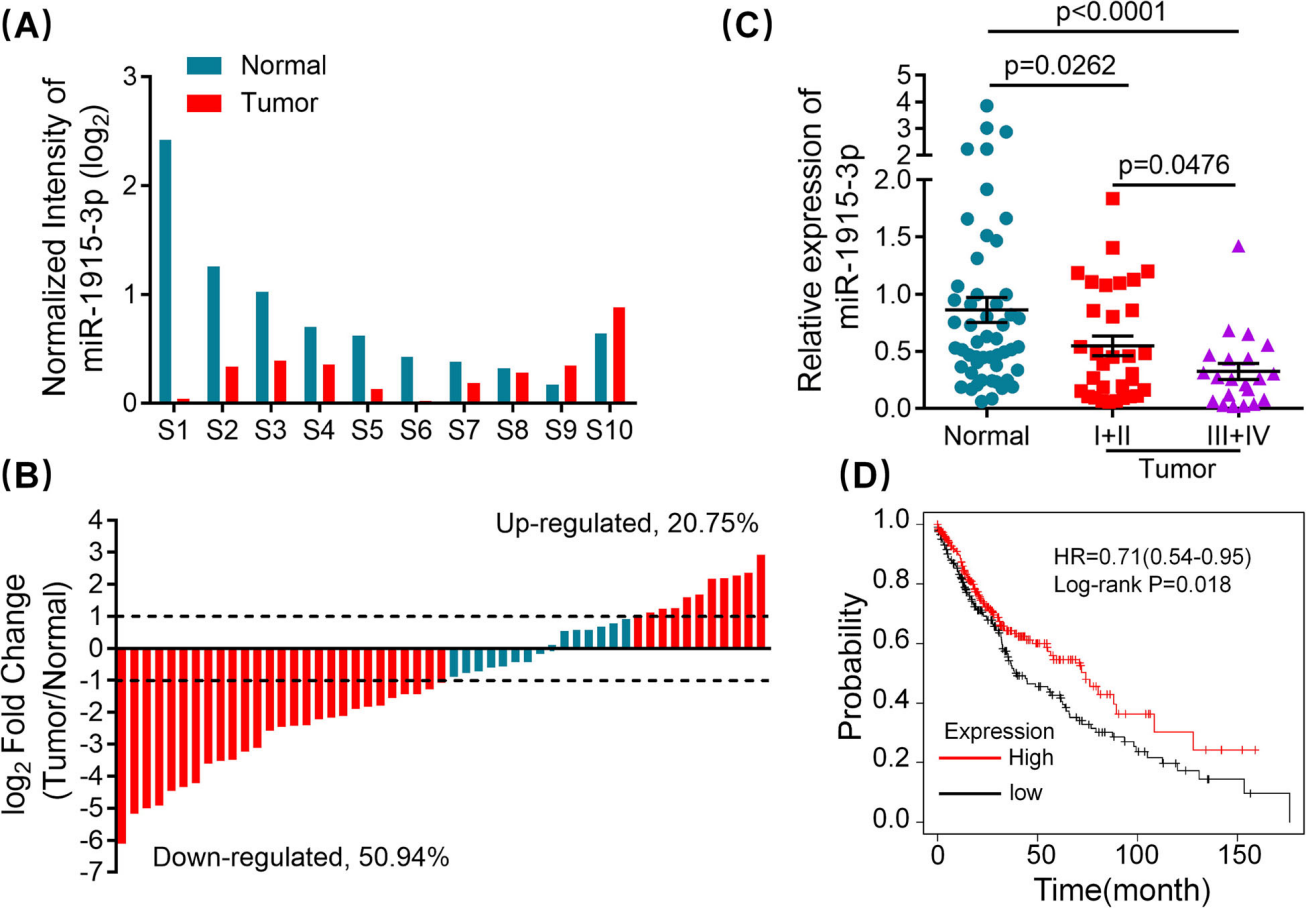
miR-1915-3p is down-regulated in NSCLC tissues and inversely correlated with the progression of NSCLC. **A** miR-1915-3p expression in 10 matched pairs of NSCLC tissues was analyzed by miRNA microarray. **B** miR-1915-3p expression was frequently decreased in NSCLC tissues. A log_2_ fold change more than + 1 or less than − 1 was considered to be significantly upregulation or downregulation, respectively. **C** NSCLC patients with lower miR-1915-3p expression had a more advanced TNM classification. **D** Kaplan-Meier survival curves of overall survival based on miR-1915-3p expression in 472 NSCLC patients. The log-rank test was conducted to compare differences between two subgroups. HR: hazard ratio

In our previous research, in order to identify differentially expressed miRNAs between NSCLC tissues and paired adjacent lung tissues, we performed miRNA microarray [16]. We found that miR-1915-3p was one of the most significantly downregulated miRNAs in NSCLC tissue samples. MiR-1915-3p expression in 8/10(80%) NSCLC patients was lower than in the paired adjacent lung tissues (Fig. 1A). Next, our results showed that miR-1915-3p expression was decreased by at least 2-fold in 32/73(43.84%) tumor tissues compared to matching normal lung samples (Fig. 1B). These patients were divided into two subgroups according to the relative expression of miR-1915-3p (log_2_ fold change of tumor/normal<0 or >0). The correlation between miR-1915-3p expression and age, gender, smoking history, histology, TNM classification, EGFR status were then analyzed. The expression of miR-1915-3p was significantly inversely correlated with clinical TNM stage (Table 2). Patients with lower miR-1915-3p expression had a more advanced TNM classification (Fig. 1C). Moreover, we further evaluated the relationship between miR-1915-3p expression and overall survival (OS) in NSCLC. Using the online bioinformatics tool Kaplan-Meier plotter [21], we found that patients with a decreased miR-1915-3p expression had much shorter overall survival compared with patients expressing increased miR-1915-3p levels (Fig. 1D). Taken together, these results revealed that miR-1915-3p was downregulated in NSCLC tissues and inversely associated with clinical TNM stage and OS, suggesting that miR-1915-3p may function as a tumor suppressor in NSCLC development.

**Table 2:** The correlation between clinical-pathological features and miR-1915-3p expression in 73 NSCLC patients

		miR-1915 Expression		
Characteristics	NO. of cases	low	high	***P*** value
Age(y)				
< 60	34	22 (64.71%)	12 (35.29%)	χ^2^ = 0.0782
≧60	39	24 (61.54%)	15 (38.46%)	*p* = 0.7798
Gender				
Male	48	31 (64.58%)	17 (35.42%)	χ^2^ = 0.1482
Female	25	15 (60.00%)	10 (40.00%)	*p* = 0.7003
Smoking history				
Yes	34	22 (64.71%)	12 (35.29%)	χ^2^ = 0.0782
No	39	24 (61.54%)	15 (38.46%)	p = 0.7798
NSCLC types				
Squamous cell carcinoma	34	19 (55.88%)	15 (44.12%)	χ^2^ = 1.3887
Adenocarcinoma	39	27 (69.23%)	12 (30.77%)	*p* = 0.2386
TNM classification				
I + II	44	22 (50.00%)	22 (50.00%)	χ^2^ = 8.0483
III + IV	29	24 (82.76%)	5 (17.24%)	*p* = 0.0046^a^
EGFR				
Del-19	18	12((66.67%)	6 (33.33%)	χ^2^ = 0.2916
L858R	12	8 (66.67%)	4 (33.33%)	*p* = 0.8643
WT	43	26 (60.47%)	17 (39.53%)	

^a^*p* < 0.05 indicated statistically significant differences. *Del-19* EGFR with a deletion in exon 19, *L858R* EGFR with L858R point mutation in exon 21, *WT* Wild-type EGFR. The Chi-square test was used to determine the correlation between miR-1915-3p expression and clinicopathological parameters

### MiR-1915-3p inhibits migration, invasion and EMT of NSCLC cells

**Fig. 2:**
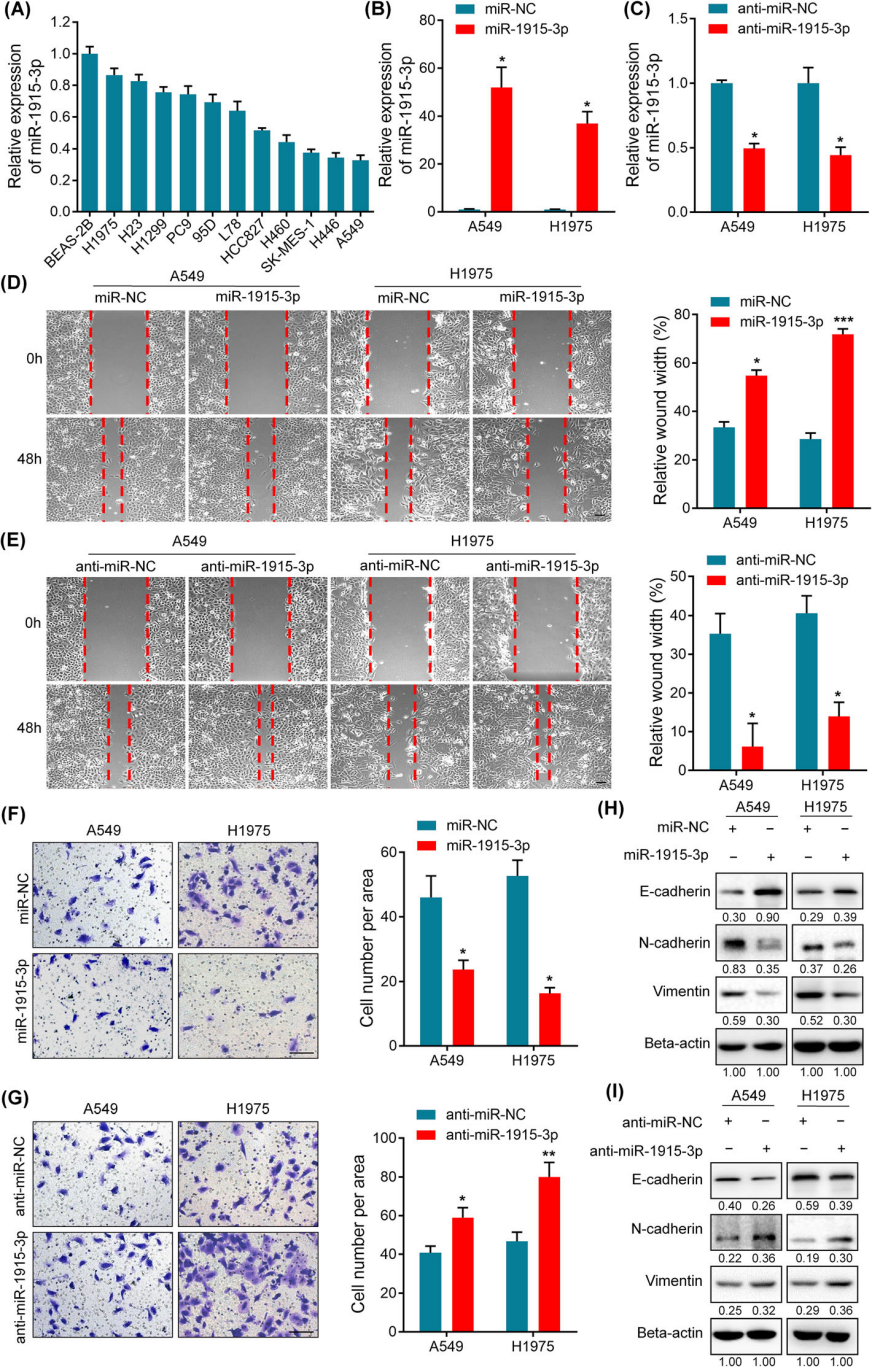
miR-1915-3p suppresses the migration, invasion and EMT of NSCLC cells. **A** miR-1915-3p expression was analyzed by qRT-PCR in eleven lung cancer cell lines and a human normal bronchial epithelial cell line. **B**, **C** miR-1915-3p expression levels in miR-1915-3p/anti-miR-1915-3p-transfected A549 and H1975 cells. **D**, **E** Wound healing assay was carried out to determine cell migratory abilities in A549 and H1975 cells transfected with miR-NC, miR-1915-3p mimic, anti-miR-NC, or anti-miR-1915-3p. Relative wound width were presented in the histogram on the right. **F**, **G** Cell invasion assay was performed in transfected-A549 and H1975 cells as described in (**D**) (**E**). The number of invading cells per area was counted. Data represent the means±SEM from three independent experiments. *p < 0.05, **p < 0.01, ***p < 0.001. Scale bars, 100 μm. (H)(I) Western blot analysis of EMT markers in miR-1915-3p/anti-miR-1915-3p-transfected A549 and H1975 cells

Considering that miR-1915-3p expression was inversely correlated with TNM stage, we speculated that miR-1915-3p might play a key role in cell migration and invasion. Initially, we examined miR-1915-3p expression levels in eleven lung cancer cell lines (H1975, H23, H1299, PC9, 95D, L78, HCC827, H460, SK-MES-1, H446 and A549) and a human normal bronchial epithelial cell line (BEAS-2B). Our data showed that the expression of miR-1915-3p was lower in lung cancer cell lines compared with normal bronchial epithelial cell (Fig. 2A). Among these lung cancer cell lines, we selected A549 (with the lowest expression of miR-1915-3p) and H1975 (with the highest expression of miR-1915-3p) for further research.

To explore the important role of miR-1915-3p in migration and invasion, a miR-1915-3p mimic or inhibitor were transfected into A549 and H1975 cells using Lipofectamine2000, respectively (Fig. 2B, C). Migration and invasion assays were then performed. These results showed that miR-1915-3p overexpression obviously suppressed migratory and invasive abilities of A549 and H1975 cells (Fig. 2D, F), whereas miR-1915-3p knockdown drastically elevated NSCLC cell migration and invasion (Fig. 2E, G).

Moreover, we tested the expression of EMT-specific markers in transfected NSCLC cells. Notably, the protein level of epithelial marker E-cadherin was up-regulated and mesenchymal markers vimentin and N-cadherin were remarkably down-regulated after miR-1915-3p overexpression (Fig. 2H). Conversely, E-cadherin was reduced and N-cadherin/vimentin were increased after miR-1915-3p silencing (Fig. 2I). These results indicated that miR-1915-3p acted as a tumor suppressor in NSCLC development by inhibiting cell migration, invasion and EMT process.

### SET is a direct target of miR-1915-3p

**Fig. 3:**
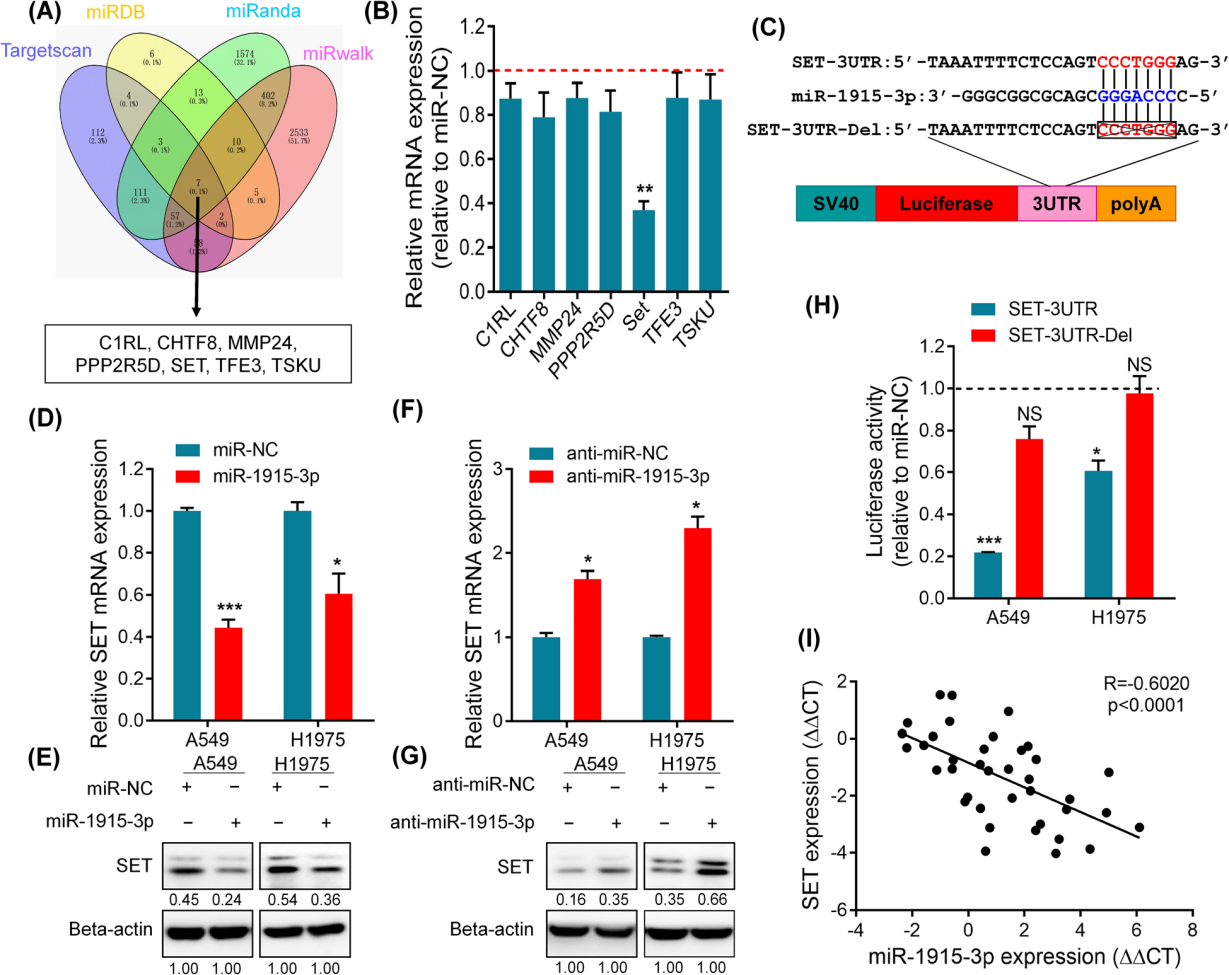
SET is a direct target of miR-1915-3p. **A** Target candidates of miR-1915-3p were predicted using four bioinformatic tools, Targetscan, miRDB, miRanda and miRwalk. **B** Relative mRNA expression of each target candidates was detected by qRT-PCR in the miR-1915-3p overexpression A549 cells. **C** Schematic depicting the construction of the WT or deletion (Del) SET 3′UTR vectors. **D**, **E** qRT-PCR and western blot analysis of endogenous SET expression in A549 and H1975 cells transfected with miR-NC or miR-1915-3p mimic. **F**, **G** Endogenous SET expression was determined both at mRNA and protein levels in anti-miR-1915-3p-transfected NSCLC cells. **H** Dual-luciferase assay was performed in A549 and H1975 cells and showed a direct binding association between SET and miR-1915-3p. Each bar represented the means±SEM for triplicate experiments. *p* values were evaluated by Student’s t-test. *p < 0.05, **p < 0.01, ***p < 0.001. NS, no significant. **I** The relationship between SET and miR-1915-3p expression was assessed by Spearman’s rank correlation analysis

To gain further insight into how miR-1915-3p regulates cell migration and invasion, we used four bioinformatic tools (Targetscan [22], miRDB [23], miRanda [24] and miRwalk [25]) to predict miR-1915-3p target candidates. As shown in the Venn diagram (Fig. 3A), seven genes (C1RL, CHTF8, MMP24, PPP2R5D, SET, TFE3 and TSKU) were identified as possible targets of miR-1915-3p. Then, mRNA levels of the seven genes were detected by qRT-PCR in the miR-1915-3p overexpressed A549 cells. We found only SET expression was dramatically suppressed among these target candidates (Fig. 3B). SET had 1 potential interacting site with miR-1915-3p (Fig. 3C). Moreover, we found that SET expression was repressed both at mRNA and protein levels when transfected with the miR-1915-3p mimics (Fig. 3D, E). In contrast, miR-1915-3p knockdown resulted in up-regulation of the SET expression (Fig. 3F, G), indicating that SET might be a target gene of miR-1915-3p.

To explore whether miR-1915-3p modulated SET expression directly, we conducted dual-luciferase assay using SET 3′UTR vector and found that relative luciferase activity was decreased by 78% in A549 cells and 39% in H1975 cells when co-transfected with miR-1915-3p mimics, which was partially alleviated when the miR-1915-3p binding site was deleted (Fig. 3H).

Furthermore, SET expression was examined in 38 NSCLC tissues and paired adjacent normal lung samples by qRT-PCR. Using spearman correlation analysis, we found that miR-1915-3p was negatively associated with the expression of SET (Fig. 3I). Thus, these results indicated that SET was a direct target gene of miR-1915-3p.

### SET is a critical mediator of miR-1915-3p effects

**Fig. 4:**
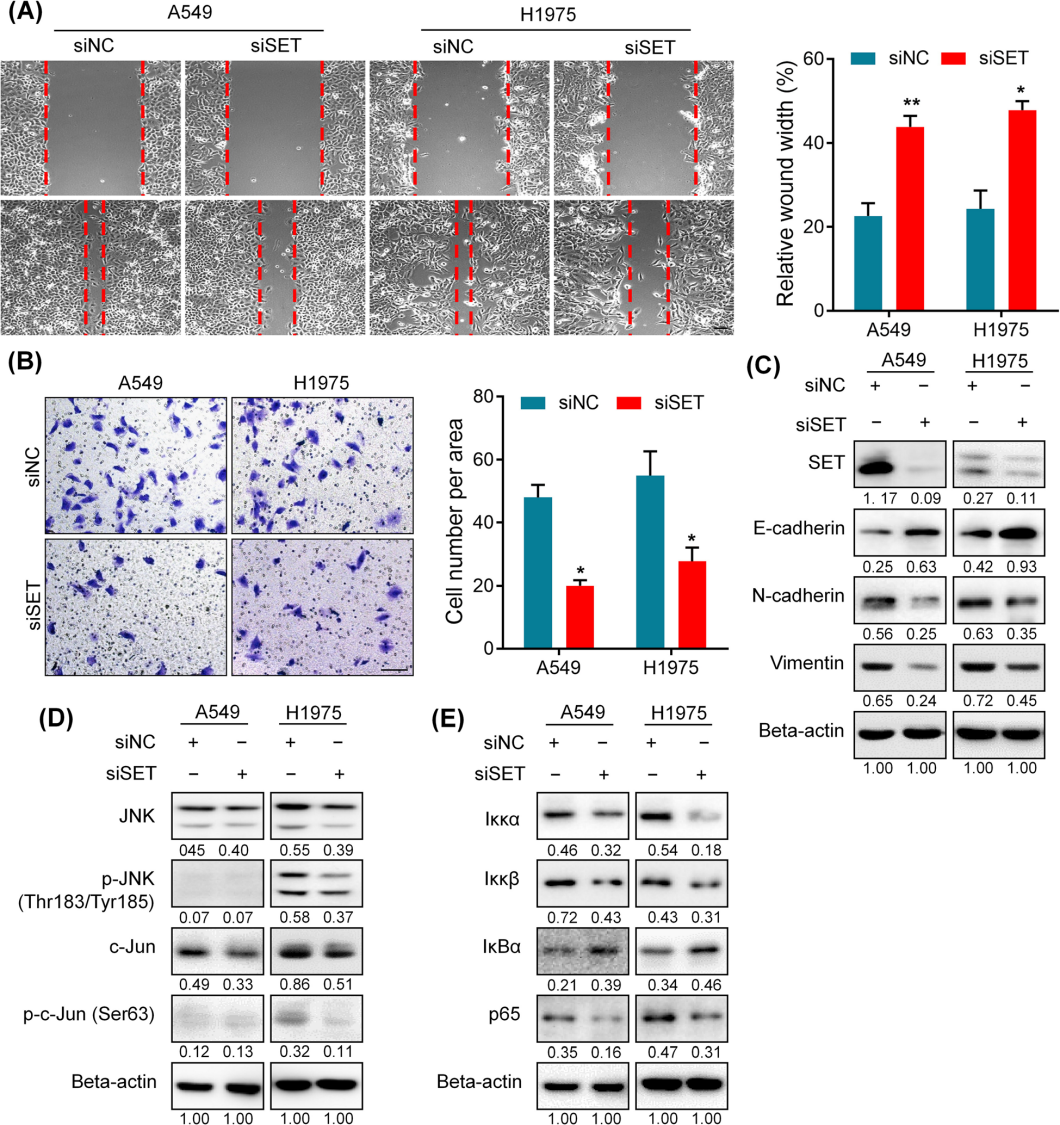
SET promotes cell migration, invasion and EMT through JNK/Jun and NF-κB signaling pathways. **A**, **B** Migration assay(**A**) and invasion assay(**B**) were conducted to determine cell migratory and invasive abilities in A549 and H1975 cells after SET silencing. **C** SET inhibition resulted in up-regulation of E-cadherin and down-regulation of N-cadherin/vimentin by western blot assay. **D**, **E** SET inhibition led to a decline of JNK/Jun and NF-κB pathway activity

To confirm whether SET inhibition recapitulated the inhibitory effect on cell migration and invasion of miR-1915-3p, we knocked down endogenous SET expression by SET siRNA (siSET) in both A549 and H1975 cells. Migration and invasion assays demonstrated that cell migratory and invasive capacities were markedly reduced after SET silence (Fig. 4A, B). Western blot assay showed that SET inhibition resulted in up-regulation of E-cadherin and down-regulation of N-cadherin/Vimentin (Fig. 4C).

Mody et al. indicated that SET up-regulated N-cadherin expression through JNK/Jun signaling pathway [26]. Therefore, we examined the protein levels of members of the JNK/Jun pathway after SET silencing. As shown in Fig. 4D, the level of p-JNK and p-c-Jun were decreased after SET silencing. In addition, previous studies found SET contributed to EMT by activating the nuclear factor-κB (NF-κB) pathway [27, 28]. We then examined the expression of proteins involved in NF-κB signaling. The level of NF-κB activation complex proteins, Iκκα and Iκκβ, were both down-regulated, while IκBα, as a NF-κB inhibitor, was significantly up-regulated when SET silenced (Fig. 4E). NF-κB p65 subunit, which plays important role in its transcription activity, was also remarkedly reduced in siSET group (Fig. 4E).

Together, the tumor suppressor effects of SET inhibition were similar to those of overexpression of miR-1915-3p. SET inhibition attenuated EMT phenotype through JNK/Jun and NF-κB signaling pathway in lung cancer cells.

**Fig. 5:**
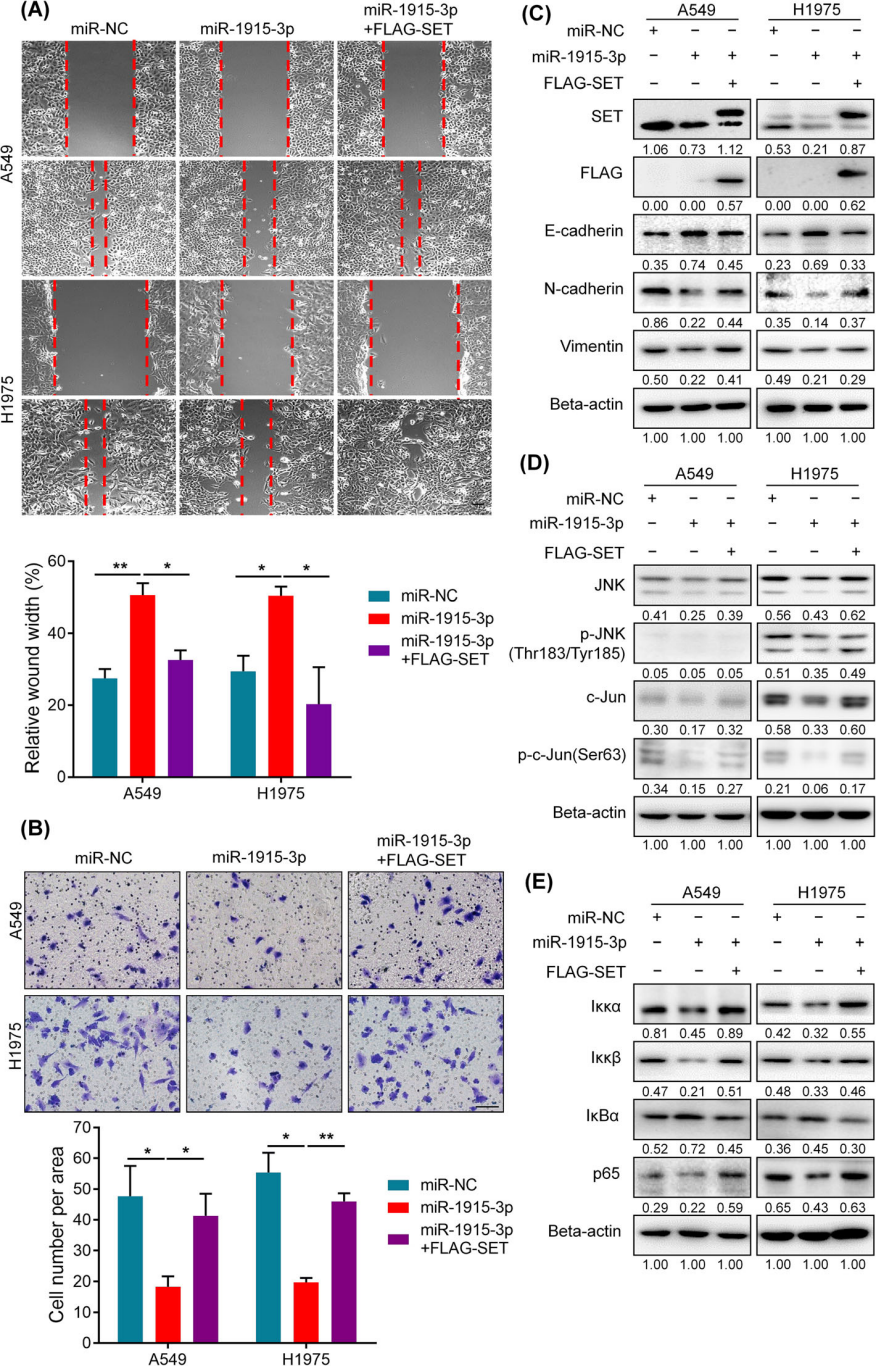
SET is a critical mediator of miR-1915-3p effects. **A**, **B** miR-1915-3p suppressed migration and invasion of NSCLC cells, and re-expression of SET rescued miR-1915-3p-induced inhibitory effects. Scale bars, 100 μm. **C** The restoration of SET expression abrogated the miR-1915-3p-mediated inhibition of N-cadherin/Vimentin and induction of E-cadherin. **D**, **E** Overexpression of miR-1915-3p alone showed a significant inhibition in JNK/Jun and NF-κB signaling pathway, whereas ectopic expression of SET attenuated these effects

Next, we further examined whether restoration of SET expression was involved in miR-1915-3p-induced suppression of NSCLC cell migration and invasion. Overexpression of miR-1915-3p alone suppressed migration and invasion of NSCLC cells, and concomitant re-expression of SET could partially overturn these effects (Fig. 5A, B). Consistent with this, the restoration of SET expression noticeably abrogated the miR-1915-3p-mediated inhibition of N-cadherin/Vimentin and induction of E-cadherin (Fig. 5C). Cells incubated with miR-1915-3p mimic alone showed a significant inhibition in JNK/Jun and NF-κB signaling pathway, whereas ectopic expression of SET attenuated these effects (Fig. 5D, E).

Collectively, our findings demonstrated that miR-1915-3p suppressed migratory and invasive capacities of NSCLC cells partly through direct targeting of SET.

### SET is highly expressed in NSCLC

**Fig. 6:**
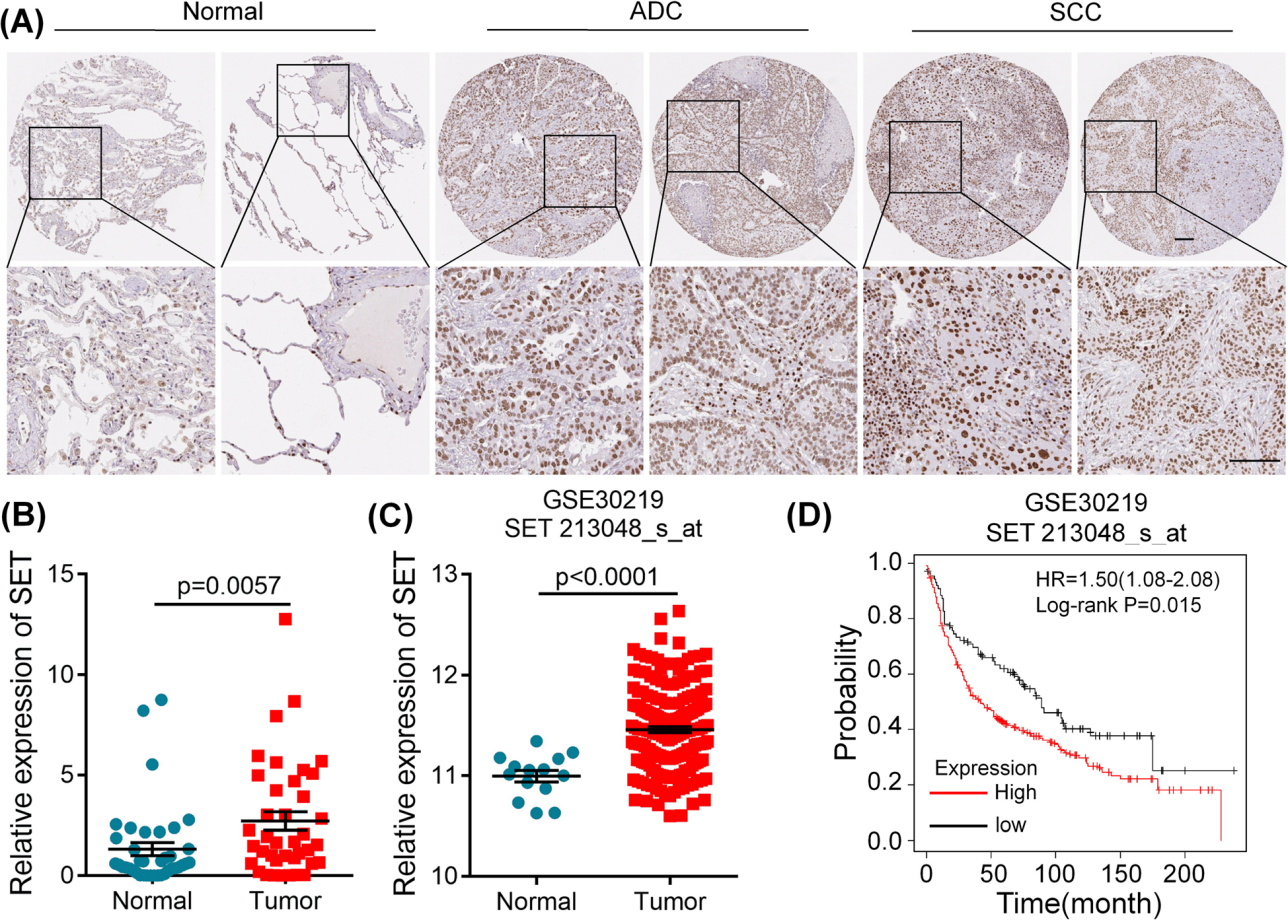
SET is highly expressed in NSCLC tissue samples. **A** SET expression was analyzed by immunohistochemistry in the human protein atlas. Scale bars, 100 μm. ADC: adenocarcinoma; SCC: squamous cell carcinoma. **B** SET mRNA expression was analyzed in 38 pairs NSCLC tissues. **C** Expression of miR-1915-3p in NSCLC and normal lung tissues calculated from GEO profiles (GSE30219, normal = 14, tumor = 202). **D** The correlation between SET expression and OS in NSCLC patients in GSE30219

To determine the expression of SET in NSCLC, the human protein atlas (https://www.proteinatlas.org/) were used to analyze SET expression at protein levels by immunohistochemistry (IHC). The IHC results showed that SET staining was strong in NSCLC specimens, but very weak in normal lung samples (Fig. 6A), suggesting that SET was highly expressed in NSCLC at protein levels. Moreover, we examined SET mRNA expression in 38 pairs NSCLC tissues and found that SET expression was significantly elevated in tumor samples (Fig. 6B). Similar results observed in GSE30219 dataset [29], NSCLC patients showed higher expression of SET as compared with normal tissues (Fig. 6C). Additionally, the correlation of SET expression with OS in GSE30219 dataset was assessed by Kaplan-Meier plotter online database. Results demonstrated that a high SET level was associated with poor prognosis (Fig. 6D).

### METTL3/KLF4 axis participate in cell migration and invasion by regulating miR-1915-3p

**Fig. 7:**
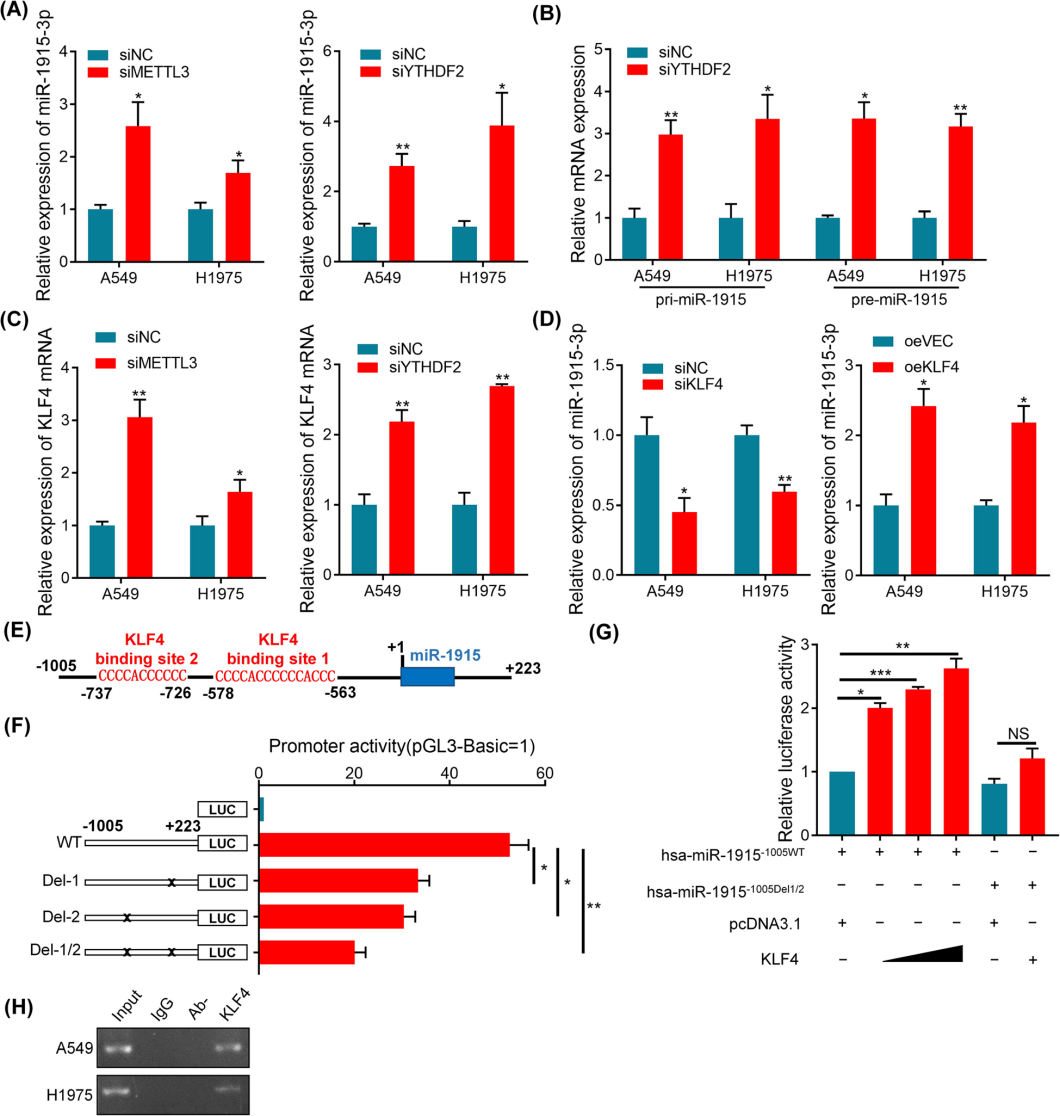
METTL3/YTHDF2 m6A axis regulates the expression of miR-1915-3p by suppressing KLF4. **A** qRT-PCR analysis of miR-1915-3p expression in A549 and H1975 cells transfected with METTL3 siRNA or YTHDF2 siRNA. **B** The relative expression of pri-miR-1915 and pre-miR-1915 were checked by qRT-PCR after YTHDF2 inhibition. **C** KLF4 expression were enhanced following METTL3 or YTHDF2 knockdown in lung cancer cells. **D** miR-1915-3p expression was analyzed in A549 and H1975 cells transfected with KLF4 siRNA or KLF4 expression vector. **E** Schematic diagram showing the KLF4-putative binding locus in the vicinity of the TSS. **F** Promoter activity of miR-1915 in A549 cells was examined using dual luciferase assays. **G** KLF4 increased the transcription activity of miR-1915 promoter, and deleting both KLF4-binding sites diminished KLF4-mediated reporter induction. **H** CHIP assays were conducted using anti-KLF4 or anti-IgG antibody. Precipitated DNA from A549 and H1975 cells was analyzed by PCR. Normal IgG and Ab-(antibody-free) were served as negative control groups, and 1% cell lysates was used as input control

In addition to the effects of miR-1915-3p on cell migration and invasion, we were also intrigued with the mechanism underlying the dysregulation of miR-1915-3p in NSCLC cells. Accumulated evidence suggested that m6A modification played crucial role in the splicing process of miRNA [30], thereby leading to dysregulation of corresponding mature miRNAs. We evaluated whether METTL3, a key m6A methyltransferase, was required in the regulation of miR-1915-3p. As illustrated in Fig. 7A, METTL3 loss significantly promoted the expression of miR-1915-3p in NSCLC cells, implying that m6A modification hindered miR-1915-3p expression. m6A modification were selectively recognized by m6A “readers” which determined the fate of m6A-modified RNA and mediated m6A function [31]. Interestingly, depletion of YTHDF2, which was an important m6A reader involved in regulation of mRNA degradation [32], could also remarkably promote miR-1915-3p expression similar to that of the METTL3 inhibition (Fig. 7A).

To further verify whether YTHDF2 mediated the biogenesis of miR-1915-3p, we then measured the levels of pri-miR-1915 and pre-miR-1915 by qRT-PCR. Our results showed that both pri- and pre-miR-1915 expression were up-regulated after YTHDF2 inhibition (Fig. 7B), indicating that YTHDF2 was not implicated in the processing of miR-1915, but might control the regulation of miR-1915-3p at transcription levels.

As previous research showed that in bladder cancer METTL3/YTHDF2 m6A axis accelerates carcinogenesis by degrading transcription factor KLF4 [33], we also examined whether METTL3/YTHDF2 m6A axis inhibited KLF4 expression in lung cancer. We found KLF4 expression were enhanced following METTL3 or YTHDF2 knockdown in lung cancer cells (Fig. 7C). In addition, the expression level of miR-1915-3p was decreased in KLF4 knockdown cells, while the level of miR-1915-3p was increased in KLF4 overexpression cells (Fig. 7D). In order to identify whether KLF4 directly promote miR-1915-3p expression by binding to miR-1915 promoter, we analyzed the genome sequence around the transcription start site (TSS) of miR-1915, noting the presence of two putative KLF4-binding sequences (CACCC) (Fig. 7E). We then constructed miR-1915 promoter reporter and deletion mutants, found that miR-1915 promoter had high transcription activity, and KLF4-binding site deletion led to a decline of transcription activity (Fig. 7F). Promoter luciferase assays indicated KLF4 increased the transcription activity of miR-1915 promoter, and deleting both KLF4-binding sites diminished KLF4-mediated reporter induction (Fig. 7G). CHIP assays using KLF4 antibody showed enrichment of miR-1915 promoter in A549 and H1975 cells, whereas negative control (IgG and Ab- groups) did not (Fig. 7H), demonstrating that KLF4 directly bound to the miR-1915 promoter in lung cancer cells and transcriptionally regulated miR-1915 expression. These results indicated that METTL3/YTHDF2 m6A axis regulated miR-1915-3p expression through transcription factor KLF4.

**Fig. 8:**
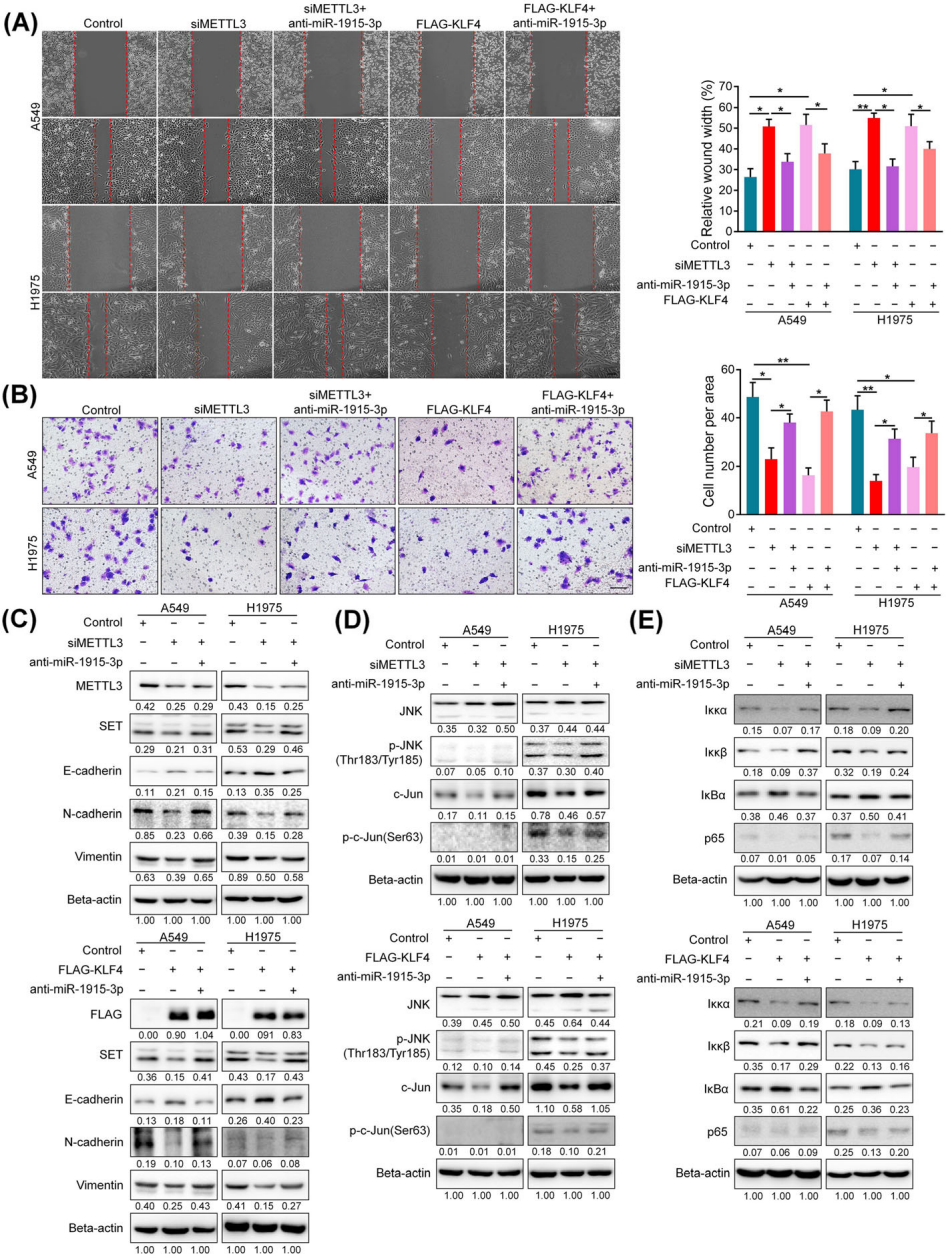
METTL3/KLF4 axis participate in cell migration and invasion by regulating miR-1915-3p. **A**, **B** Migration assay(**A**) and invasion assay(**B**) were conducted to determine cell migratory and invasive abilities in A549 and H1975 cells after METTL3 silencing or KLF4 overexpression. Scale bars, 100 μm. **C**, **D**, **E** Western blot analysis for related proteins that were involved in EMT, JNK/Jun and NF-κB pathways

Next, we further examined the effects of METTL3/KLF4 axis on cell migration and invasion. We found that METTL3 silencing or KLF4 overexpression could suppress cell migration and invasion, and concomitant miR-1915-3p inhibition partially overturn these effects (Fig. 8A, B). Consistent with this, METTL3 silencing or KLF4 overexpression inhibited EMT, JNK/Jun and NF-κB signaling pathways, whereas miR-1915-3p inhibition attenuated these effects (Fig. 8C, D, E).

## Discussion

Lung cancer is the second most commonly diagnosed cancer with high fatality in the world. Increasing evidences have shown that miRNAs play significant roles in carcinogenesis and development of NSCLC.

In this report, our results revealed that miR-1915-3p was markedly repressed in NSCLC cell lines and tissues and that down-regulation of miR-1915-3p was inversely associated with clinical TNM stage and overall survive, suggesting that miR-1915-3p may function as a tumor suppressor in NSCLC development. Similarly, previous results showed that the relative expression of miR-1915-3p was down-regulated in gastric cancer and modulated the development of gastric cancer through the repression of RAGE and BCL-2 [19, 34]. Down-regulation of miR-1915-3p was responsible for multidrug resistance in colorectal cancer, and re-establishing miR-1915-3p could restore the chemosensitivity depend on suppression of BCL-2 and NFIX expression [17, 35]. Jin et al. demonstrated that there were low levels of miR-1915-3p in breast cancer, which inhibited H3K4 methylation, proliferation and migration by targeting SETD1A [36]. Consistent with our findings, these results indicated that miR-1915-3p functioned as a tumor suppressor and blocked tumor progression by regulating several signaling pathways. However, opposite results are also obtained by some previous researches. Wan and colleagues showed that miR-1915-3p was upregulated by p53-dependent pathway under oxidative stress in hepatocellular carcinoma [37]. In thyroid carcinomas, high expression levels of miR-1915-3p associated with infiltrative growth of follicular variant of papillary thyroid carcinomas [38]. Guo et al. reported that miR-1915-3p was markedly higher in breast cancer patient’s serum and associated with lymph node metastasis. Ectopic expression of miR-1915-3p enhanced cell proliferative and migratory capacities by repressing of DUSP3 and activation of ERK1/2 signaling [39]. In addition, miR-1915-3p could impair etoposide-induced apoptosis, thereby increasing chemoresistance via downregulating DRG2 and PBX2 in lung cancer cell lines (NCI-H441 and NCI-H1650) [40]. These divergent results might be due to the differences in various cancers, cell types or tumor heterogeneity. In the present study, we found that miR-1915-3p inhibited migration, invasion and EMT of NSCLC cells, acting as a tumor suppressor. Through bioinformatic analysis, SET was predicted as a potential target gene of miR-1915-3p. Then the dual-luciferase reporter assay confirmed that miR-1915-3p could directly interact with SET 3′UTR, thereby depressing the expression of SET in NSCLC cells. Therefore, our results demonstrated that miR-1915-3p enforced its anti-oncogenic role through directly targeting SET, and restoration of miR-1915-3p in NSCLC may hold therapeutic promise.

SET, an endogenous inhibitor of PP2A, plays a pivotal oncogenic role in tumorigenesis [41]. SET has been identified as an oncogene in multiple types of cancers, such as hepatocellular carcinoma [42], pancreatic cancer [26], glioblastoma multiforme [43] and lung cancer [44, 45]. In the current study, we confirmed that SET was substantially increased in NSCLC specimens and associated with worse clinical outcome, which was consistent with previous studies [44, 45]. In addition, previous studies showed that SET could promote EMT and metastasis by modulating several signaling pathways. Liu et al. concluded that SET could depress the expression of NDRG1 through PP2A/c-MYC pathway, resulting in activation of EMT process, whereas blockade of SET by FTY720 (fingolimod) could inhibit EMT and restore the chemosensitivity in A549 lung cancer cells [27]. NDRG1 upregulated E-cadherin via NF-κB signaling pathway [28]. Mody et al. suggested that SET mediated EMT transition by promoting N-cadherin expression through Rac1/JNK/c-Jun signaling pathway in pancreatic cancer [26]. Herein, our findings indicated that knockdown of SET led to repression of migration and invasion, upregulation of epithelial marker (E-cadherin), and downregulation of mesenchymal markers (N-cadherin/Vimentin) in NSCLC cells, which recapitulated miR-1915-3p-induced inhibitory effects on cell migration, invasion and EMT process through JNK/c-Jun and NF-κB signaling pathways. Furthermore, we verified that miR-1915-3p controlled cancer migration, invasion and EMT process through directly downregulating SET in lung cancer cells.

M6A marks have been identified as a key regulator of miRNA biogenesis by suppressing the binding of DGCR8 to pri-miRNAs [46]. Aberrant expression of METTL3 may be significantly responsible for the alteration of miRNAs. It has been reported that METTL3 modulated the pri-miR221/222 and pri-miR1246 process in a DGCR8-dependent manner [47, 48]. In addition, previous study indicated that METTL3 could facilitate the cleaving of miR-143 precursor to form mature miRNA in lung cancer [49]. Meanwhile, Alarcon et al. have shown that m6A “reader” protein HNRNPA2B1 can directly bind to m6A marks of primary miRNA transcripts to modulate miRNA biogenesis [50]. Our present study pointed out that METTL3 loss significantly promoted the expression of miR-1915-3p, whereas HNRNPA2B1 had no effect on the expression of miR-1915-3p (data not show). However, depletion of m6A “reader” protein YTHDF2 could promote miR-1915-3p expression and result in accumulation of pri/pre-miR-1915, indicating that YTHDF2 was not involved in the processing of miR-1915, and might control miR-1915-3p expression through transcriptional regulation. Xie and colleagues have determined that the METTL3/YTHDF2 m6A axis directly degraded the mRNA of the transcription factor KLF4, contributing to the progression of bladder cancer [33]. Subsequently, we validated that METTL3/YTHDF2 m6A axis also repressed KLF4 expression in lung cancer. Furthermore, KLF4 directly bound to the miR-1915 promoter and transcriptionally regulated miR-1915 expression in lung cancer cells. METTL3 silencing or KLF4 overexpression could suppress cell migration and invasion, and concomitant miR-1915-3p inhibition partially overturn these effects. These results indicated that METTL3/KLF4 axis participated in cell migration and invasion by regulating miR-1915-3p.

## Conclusion

Collectively, we identified that miR-1915-3p, as a crucial tumor suppressor, was significantly down-regulated in NSCLC, and inversely associated with clinical TNM stage and overall survival. Functional studies indicated that miR-1915-3p suppressed cancer migration, invasion and EMT of NSCLC via directly targeting oncogene SET. In addition, the expression of miR-1915-3p was regulated by METTL3/YTHDF2 m6A axis through transcription factor KLF4. These findings that the dysregulation of miR-1915-3p was involved in NSCLC metastasis indicate that miR-1915-3p may have an anti-metastatic therapeutic potential for lung cancer treatment.

## Data Availability

The datasets used and/or analyzed during the current study are available from the corresponding author on reasonable request.

## Abbreviations

miRNA: microRNAs
NSCLC: Non-small-cell lung cancer
qRT-PCR: Quantitative real-time PCR
CHIP: Chromatin immunoprecipitation
EMT: Epithelial-mesenchymal transition
PROM1: Prominin 1
PAX2: Paired box 2
